# Design and Characterization of Epoxy/Graphite Flake Composites for Enhanced Electrical Conductivity and Electrochemical Performance in Energy Storage Applications

**DOI:** 10.3390/polym18040502

**Published:** 2026-02-17

**Authors:** Saleh R. Al-Bashaish, Anas Y. Al-Reyahi, Milica Vujković, Tamara Petrović, Ivan Petronijević, Slavica Maletić, Rashid Dallaev, Ammar Alsoud, Dinara Sobola

**Affiliations:** 1 Department of Allied Sciences, Faculty of Arts and Sciences, Al-Ahliyya Amman University, Amman 19328, Jordan; s.albashaish@ammanu.edu.jo; 2 Department of Physics, Faculty of Science, The Hashemite University, Zarqa 13133, Jordan; 3 Faculty of Physical Chemistry, University of Belgrade, Studentski trg 12-16, 11000 Belgrade, Serbia; 4 Faculty of Physics, University of Belgrade, Studentski trg 12-16, 11000 Belgrade, Serbia; ivanpetronijevic@ff.bg.ac.rs (I.P.);; 5 Department of Physics, Faculty of Electrical Engineering and Communication, Brno University of Technology, Technická 2848/8, 61600 Brno, Czech Republic; 6 Institute of Physics of Materials, Czech Academy of Sciences, Žižkova 22, 61662 Brno, Czech Republic

**Keywords:** epoxy-graphite composites, electrical conductivity enhancement, energy storage, electrochemical performance, flake dispersion optimization

## Abstract

This study presents a comprehensive investigation of the electrical, structural, and electrochemical properties of graphite flake (GF)-reinforced epoxy composites for energy storage applications. Epoxy/GF composites with filler loadings of 10, 30, 50, 70, and 80% wt. were fabricated to evaluate the effect of graphite concentration on conductivity, charge storage, and structural integrity. Impedance spectroscopy demonstrated that quantum-mechanical tunneling, consistent with fluctuation-induced tunneling transport, predominates charge transfer over a wide temperature range, ensuring strong electrical performance. The results show that at 10–30% wt.% GFs, incomplete conductive networks and limited electron and ion transport reduce electrochemical performance. At 50–70% wt.% GFs, the composites exhibited the highest specific capacitance and excellent cyclic stability due to the formation of well-connected three-dimensional conductive networks with sufficient porosity for efficient ion diffusion and charge transport. At filler loadings above 70 wt.%, graphite agglomeration, pore blockage, and microstructural defects were observed, resulting in reduced conductivity and capacitance. SEM, FTIR, and XRD analyses confirmed optimal chemical and morphological interactions at moderate filler contents, highlighting structural degradation at excessive loadings. These results indicate that an optimal graphite content of 50–70% by weight balances conductive pathways, mechanical stability, and electrolyte accessibility, providing a blueprint for designing epoxy/graphite composites that are robust and efficient for next-generation energy storage devices.

## 1. Introduction

Conductor–insulator composites constitute a technologically significant class of hybrid materials, synthesized by embedding electrically conductive fillers—such as carbon-based particles, metallic nanomaterials, or conductive polymers—within an insulating matrix (e.g., polymers or ceramics). These composites exploit the complementary properties of their constituents: the insulating matrix ensures mechanical robustness, thermal stability, and ease of processing, while the conductive phase tailors electrical conductivity and functional performance [1,2]. A pivotal characteristic of such systems is the percolation threshold, the critical filler concentration at which a continuous conductive network forms, enabling a transition from insulating to conductive behavior. By strategically optimizing filler type, concentration, spatial distribution, and interfacial interactions, these materials achieve tunable electrical, thermal, and mechanical properties, rendering them vital for cutting-edge applications [3]. For instance, they are widely employed in electromagnetic interference (EMI) shielding [4], flexible strain sensors [5], anti-static coatings [6], and high-efficiency energy storage devices [7]. The unique capacity of conductor–insulator composites to merge lightweight design, cost-effectiveness, and multifunctionality—while circumventing the limitations of purely conductive or insulating materials—positions them as transformative solutions for next-generation technologies [8,9,10,11].

GFs-based composites have garnered significant attention in recent years for their exceptional electrical and economic advantages [12]. These composites exhibit a low percolation threshold, enabling high electrical conductivity across broad frequency ranges, including the microwave and terahertz regimes, which is critical for advanced electromagnetic applications [13]. Furthermore, graphite flakes offer a cost-effective alternative to other carbon allotropes, such as carbon black or carbon nanotubes (CNTs), while avoiding the toxicity concerns associated with CNTs. The potential health and environmental risks of CNTs have been extensively debated in previous studies [14,15,16] and remain unresolved, underscoring the safety benefits of graphite-based composites. Combined with their non-toxic profile and competitive pricing, these attributes position graphite flake composites as a sustainable and scalable solution for next-generation conductive materials.

Composite materials incorporating graphene-filled epoxy resin, GFs, or their functionalized derivatives have been widely explored in recent research due to their exceptional multifunctional properties. Yao et al. [17] investigated the lithium-ion conductivity and electrochemical behavior of a rigid random copolymer (PGO) synthesized from glycidyl methacrylate (GMA) and singlet oxygen (OE). The study focused on GMA due to its highly reactive epoxy moieties, which enable versatile post-polymerization functionalization, and OE, which introduces tailored structural properties. The rigidity of the copolymer, evidenced by the relatively high glass transition temperature (T = 68 °C) of the PGMA homopolymer, was found to facilitate stable film formation—a critical requirement for solid-state electrolyte applications. However, the influence of the polar epoxy groups on lithium-ion transport mechanisms remains unresolved, presenting a key research gap. Rus A et al. [18] systematically evaluated the influence of graphite filler incorporation (up to 30 wt.%) on the mechanical performance of cast bio-based epoxy composites. The study employed detailed morphological characterization, including scanning electron microscopy (SEM) and interfacial analysis, to elucidate the dispersion homogeneity and bonding efficacy of graphite particles within the epoxy matrix. Results revealed that achieving uniform dispersion of the filler posed significant challenges, with agglomeration observed at higher loadings (>20 wt.%), which directly correlated with diminished tensile strength and fracture toughness. These findings underscore the critical role of optimized processing protocols in mitigating filler aggregation and enhancing interfacial adhesion, particularly in sustainable composite systems targeting structural applications.

This study addresses these challenges by designing epoxy composites with high filler loadings (10–80 wt.%), aiming to form percolating conductive networks while preserving structural integrity. Electrical conductivity was investigated over a wide frequency range (10^2^–10^6^ Hz) and elevated temperatures (30–170 °C), providing insights into charge transport mechanisms, activation energies, and thermal stability. Cyclic capacitance and voltage tests further demonstrated robust energy storage capability. The work demonstrates that ultrahigh-filler-content epoxy composites can achieve stable conductivity under harsh conditions while remaining scalable and eco-friendly, highlighting their potential as sustainable materials for next-generation energy storage applications.

## 2. Sample Preparation

**Figure 1:**
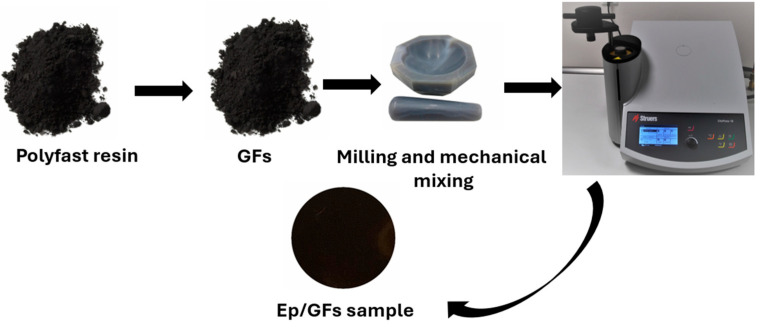
The synthesis process of the Ep/GFs composite.

A poly-fast resin-type conductive epoxy (powder form) from Struers (Ballerup, Denmark) was used as the matrix due to its compatibility with filler integration. High-purity GFs (99.5%, thickness ~100 nm, lateral size ~10 µm) from Sigma-Aldrich (St. Louis, MO, USA) were incorporated into the epoxy at concentrations of 10, 30, 50, 70, and 80 wt.%. The total sample mass was 0.2 g. The components were precisely weighed and manually mixed with sufficient force, which partially softened the epoxy matrix and improved its adhesion to the GF. The mixture was then thermally cured at 180 °C under 350 Pa for 3 min, followed by a 5 min cooling phase, using an automated CitoPress compounding press (Struers, Rotherham, UK). The resulting samples had a radius of 3 cm and a thickness of 150 µm, measured with an electronic Vernier Caliper to ensure accuracy. The synthesis process is illustrated in Figure 1.

## 3. Characterization

### 3.1. SEM Micrograph

**Figure 2:**
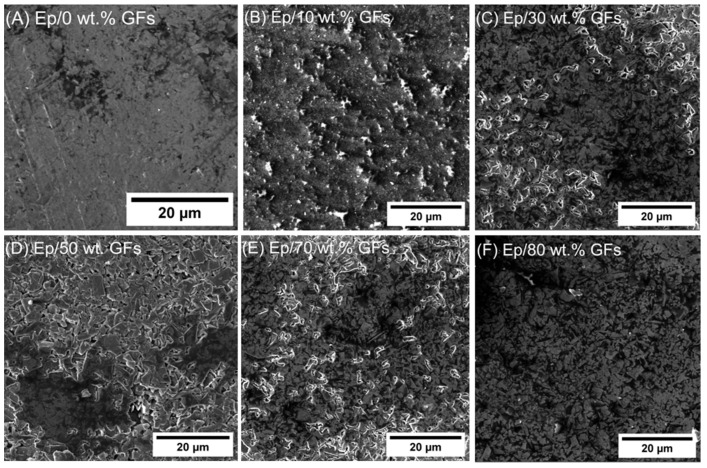
SEM micrograph for (**A**) Ep/0 wt.% GFs. (**B**) Ep/10 wt.% GFs (**C**) Ep/30 wt.% GFs. (**D**) Ep/50 wt.% GFs. (**E**) Ep/70 wt.% GFs. and (**F**) Ep/80 wt.% GFs.

Figure 2 presents SEM images of Ep/GFs composites across a wide filler concentration range of 0–80 wt.%. The unfilled epoxy matrix exhibits a smooth, non-featured surface, serving as a reference for morphological comparison. At low filler loadings (10–30 wt.%, Figure 2B,C), the GFs are relatively well dispersed within the polymer matrix, although significant epoxy-rich regions persist between the flakes. This microstructure results in incomplete conductive networks that limit electronic conduction and ionic access, which negatively affects electrochemical performance. At intermediate concentrations (50–70 wt.%, Figure 2D,E), a clear microstructural transition occurs. The composites exhibit three-dimensionally interconnected graphite networks, well-distributed mesoporous channels, and strong interfacial adhesion between the filler and matrix. This architecture enhances both electron transport and electrolyte penetration, supporting efficient charge storage and transport, which explains the observed peak in electrochemical performance.

At high filler loading (80 wt.%, Figure 2F), the microstructure shows notable degradation. Localized graphite agglomerates form microscale clusters, mesoporous channels become partially obstructed, and the tortuosity of both electronic and ionic transport pathways increases. These changes reduce the accessible surface area, introduce electron scattering at agglomerate boundaries, and create isolated epoxy domains, collectively leading to a decline in performance despite the higher filler content.

### 3.2. FTIR

**Figure 3:**
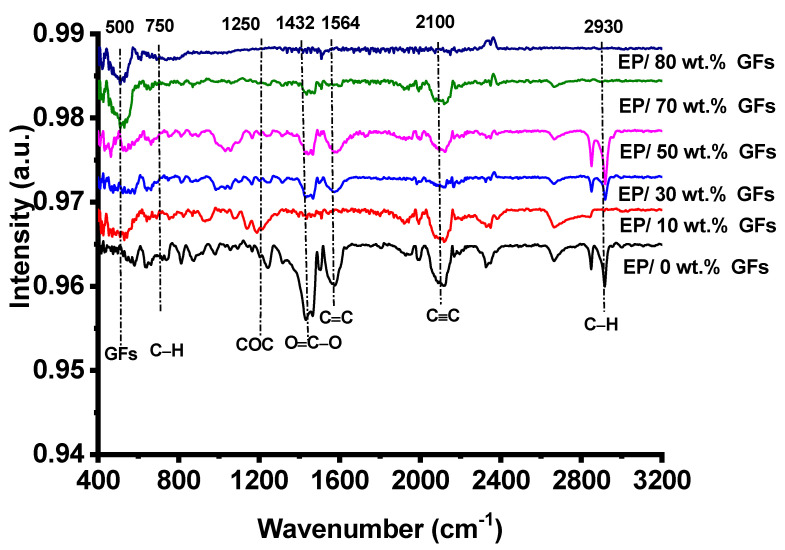
FTIR spectra of unfilled and Ep/GFs composites.

FTIR analysis provides detailed information on the structure-property relationships governing the performance of epoxy/graphite composites. At low filler concentrations (10–30 wt.%), the spectra show attenuated O–H stretching vibrations at 3400 cm^−1^ and intact C=O bonds at 1700 cm^−1^, indicating limited chemical interactions between the GFs and the epoxy matrix, which is consistent with morphological observations of discontinuous conductive networks and restricted ionic accessibility that can hinder electrochemical performance [19]. At intermediate loadings (50–70 wt.%), the FTIR spectra reveal the emergence of C=C stretching vibrations around 1600 cm^−1^ and noticeable shifts in the hydroxyl peaks, reflecting enhanced interfacial bonding, improved filler-matrix adhesion, and maintained structural integrity, thus supporting efficient charge transport and enhanced electrolyte accessibility that explain the peak electrochemical performance. At high filler loading (80 wt.%), significant chemical changes are observed, including the disappearance of the C–O–C ether linkages at 1250 cm^−1^ and broadening of absorption bands, indicating the disintegration of the polymer matrix [20]. Chemical degradation reduces the load-transfer capacity of the epoxy matrix, facilitating microcrack formation and increasing charge-transport tortuosity at elevated filler concentrations. This chemical deterioration is consistent with SEM observations of increased transport tortuosity, localized graphite agglomeration, and isolated epoxy domains, all of which reduce electron and ion transport [21]. The concentration-dependent structural evolution of the composites is illustrated in Figure 3, highlighting the critical role of filler content in balancing chemical integrity and electrochemical functionality.

### 3.3. XRD

**Figure 4:**
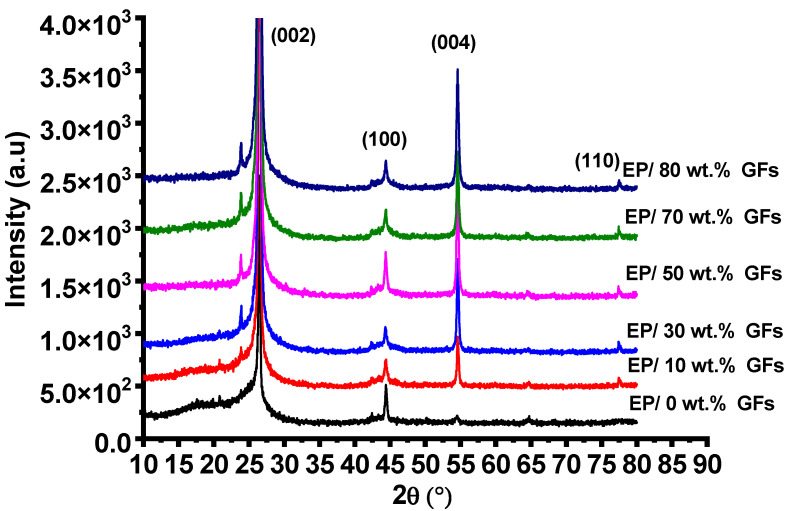
XRD pattern for Ep/GFs composites.

The XRD patterns of the EP/GFs composites are presented in Figure 4. The results indicate that the epoxy matrix is semi-amorphous (amorphous), which is consistent with previous reports in the literature [1]. The unfilled epoxy matrix exhibits two main diffraction peaks at 2θ ≈ 26° and 44°, along with weaker reflections at approximately 55° and 65°, indicating the presence of carbon-related structural features. The prominent (002) diffraction peak at 2θ = 26.6° corresponds to an interplanar spacing (d002) of 0.334 nm, which is characteristic of the layered structure of graphite. It should be noted that a broad feature around 2θ ≈ 23° is sometimes observed in carbon-based materials, reflecting slight disorder or partial stacking defects in graphite flakes. This peak is distinct from the (002) graphite peak and helps to account for minor structural irregularities in the GFs used [2,3].

The prominent (002) diffraction peak observed at 2θ = 26.6° corresponds to an interplanar spacing (d002) of 0.334 nm, which is characteristic of the layered structure of graphite. Additional diffraction peaks at 43.3° (corresponding to the (100) plane), 54.6° (attributed to the (004) plane), and 77.8° (assigned to the (110) plane) further confirm the crystalline graphite structure. These results are in good agreement with previously reported studies [22,23].

## 4. Results and Discussion

### 4.1. Conductivity

**Figure 5:**
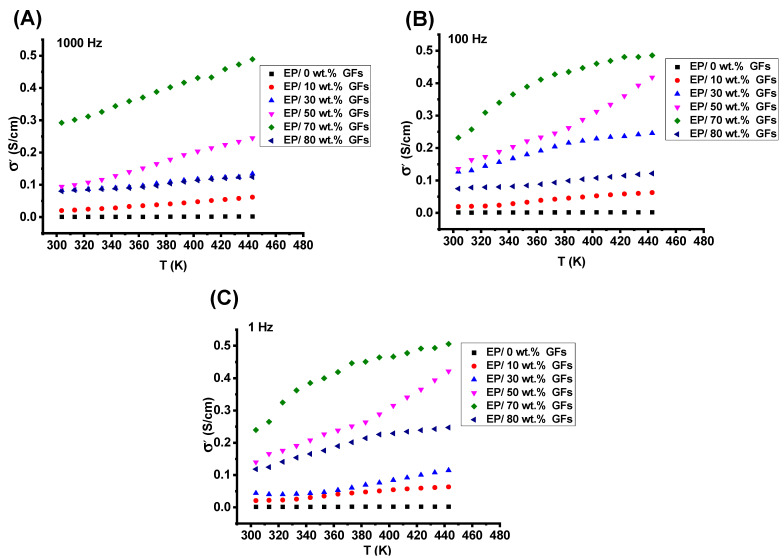
Temperature dependence of AC real electrical conductivity (σ′) at different frequencies: (**A**) 1000 Hz, (**B**) 100 Hz, and (**C**) 1 Hz.

Figure 5A–C present the variation in the real electrical conductivity (σ′) with temperature for neat epoxy and epoxy/graphite flake (Ep/GFs) composites in the frequency range of 1 Hz to 1 kHz. As the graphite content increases from 10 wt.% to 80 wt.%, a gradual enhancement in σ′ is observed. This improvement is attributed to the formation of a well-connected three-dimensional conductive network that enables efficient charge transport via Ohmic conduction through direct graphite–graphite contacts and tunneling across the insulating epoxy layers. At these filler concentrations, the internal porosity remains relatively low, and the conductive pathways remain open and interconnected, allowing electrons to move with minimal hindrance.

However, when the filler loading exceeds 70 wt.%, σ′ deteriorates with increasing temperature. This decline is explained by increased porosity from excessive graphite packing, which blocks fine pores and disrupts certain conductive pathways [24]. Furthermore, the sharp reduction in the epoxy fraction at such high loadings diminishes the matrix’s ability to absorb mechanical and thermal stresses, increasing brittleness and further limiting efficient charge transport [24].

Above the percolation threshold, electronic conduction dominates the system’s dielectric response, overshadowing dipolar relaxation effects. As temperature rises, thermal fluctuations significantly decrease the tunneling barrier height at graphite–epoxy interfaces, thereby increasing the carrier transmission probability without requiring structural rearrangements [1]. In contrast, neat epoxy exhibits frequency-dependent behavior with negligible temperature influence, indicating the absence of thermally activated charge carriers [25].

**Figure 6:**
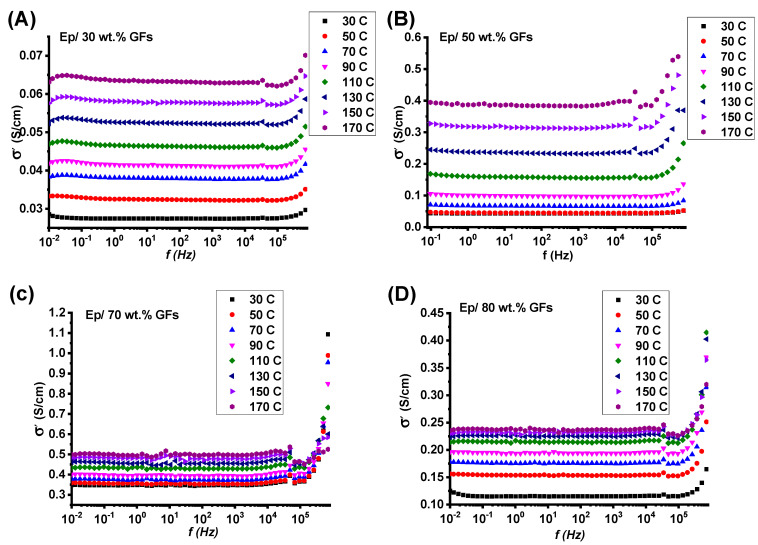
Frequency-dependent conductivity for (**A**) Ep/30 wt.% GFs, (**B**) Ep/50 wt.% GFs, (**C**) Ep/70 wt.% GFs. (**D**) Ep/80 wt.% GFs.

Figure 6A–D present the frequency-dependent conductivity spectra of Ep/GF composites across a filler loading range of 30–80 wt.%. The spectra reveal three characteristic regions: a low-frequency DC plateau (below 10^5^ Hz), where $\sigma_{DC}$ progressively increases with graphite content up to 70 wt.%; a transition region marking the onset of frequency-dependent behavior; and a high-frequency dispersion regime (above 10^5^ Hz) dominated by AC conduction mechanisms. The 50 wt.% and 70 wt.% composites exhibit the most continuous and highly interconnected conductive pathways, corresponding to the highest conductivity values. In contrast, the 80 wt.% composite shows a notable reduction in conductivity despite its higher filler content. This reversal is attributed to excessive filler packing, which generates tortuous and meandering conduction pathways, increases interfacial scattering sites, and introduces structural defects that partially disrupt the percolation network.

The crossover frequency $\left(f_{c}\right)$ between the DC and AC regimes shifts systematically with filler concentration, reflecting the evolution of the conductive network from isolated clusters (30 wt.%) to fully developed percolation (70 wt.%), and finally to an oversaturated, defect-limited system at 80 wt.% [26].

**Figure 7:**
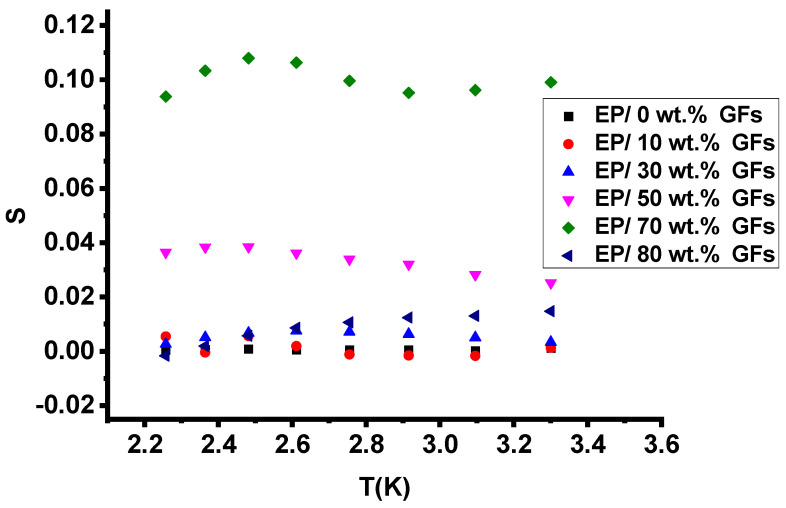
Temperature dependence of the exponent s.

**Figure 8:**
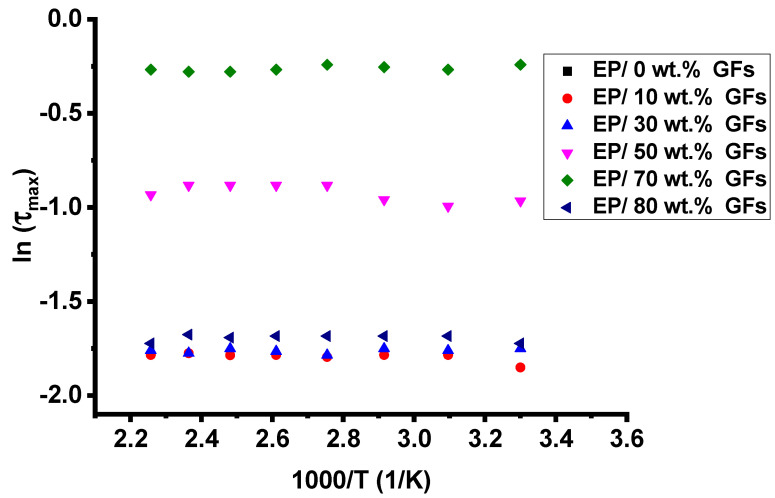
Temperature dependence of the exponent $\tau_{max}$.

Figure 7 shows the temperature dependence of the frequency exponent (s) for Ep/GFs composites, which remains nearly constant, indicating that quantum-mechanical tunneling (QMT) dominates charge transport. Figure 8 shows ln(τ_max_) versus 1000/T, with a slope close to zero, indicating a negligible activation energy (E_a_) and minimal contribution from thermally activated processes such as hopping conduction or ionic mobility. This behavior is consistent with the fluctuation-induced tunneling (FIT) concept, in which charge carriers tunnel through thin insulating epoxy barriers separating adjacent graphite flakes, resulting in a weak temperature dependence of conductivity and a practically constant frequency exponent [27].

This temperature-independent transport has important implications for energy storage applications. The dominance of QMT and FIT ensures efficient electron transport across the epoxy barriers, providing stable conductivity over a wide temperature range. In supercapacitors, this enables rapid charge/discharge cycles with low internal resistance and minimal energy loss. At the same time, in battery electrodes, it contributes to higher power density and reliable operation under varying environmental conditions.

In these systems, charge carriers traverse the insulating epoxy barriers between adjacent graphite flakes via temperature-independent quantum tunneling, which depends on the spatial proximity of the fillers and the potential barrier height. The sub-percolative microstructure, with homogeneous dispersion of graphite flakes and optimized interfacial spacing, enhances tunneling efficiency and ensures stable electrical conductivity over a wide temperature range (30–170 °C). These characteristics make Ep/GFs composites promising for high-performance energy storage and electronic devices that require thermal stability.

### 4.2. Electrochemical Measurements

Cyclic voltammetry (CV) measurements of epoxy/graphite flake (Ep/GFs) composites were conducted using a standard three-electrode cell comprising a platinum counter electrode (Pt), a saturated calomel electrode (SCE) as the reference, and the modified composite as the working electrode. The measurements were performed in 1 M aqueous H_2_SO_4_ at room temperature with scan rates of 20 and 50 mV s^−1^. The specific capacitance values were calculated from the CV curves using the integrated current response, accounting for the active material mass, the scan rate, and the applied potential window.

**Figure 9:**
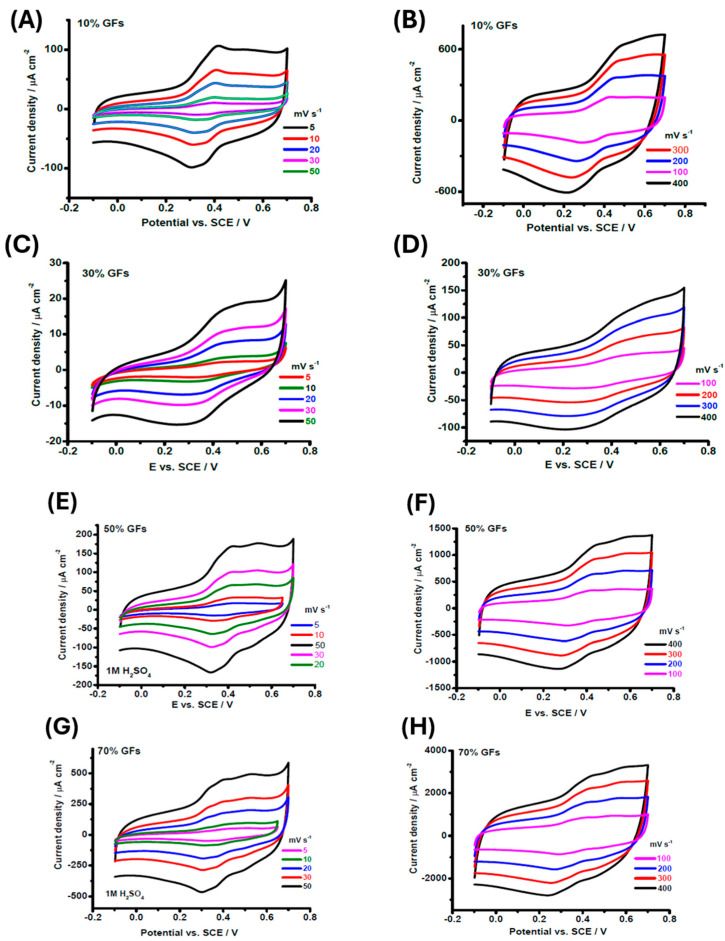

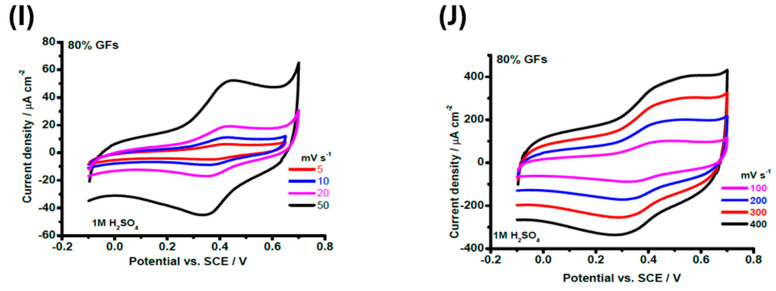
CV curves of EP/GFs composites during the initial cycling in 1 M H_2_SO_4_ at different scan rates. (**A**,**B**) EP/10 wt.% GFs; (**C**,**D**) EP/30 wt.% GFs; (**E**,**F**) EP/50 wt.% GFs; (**G**,**H**) EP/70 wt.% GFs; (**I**,**J**) EP/80 wt.% GFs. Low scan rates (5–50 mV s^−1^) are shown in (**A**,**C**,**E**,**G**,**K**), and high scan rates (100–400 mV s^−1^) are shown in (**B**,**D**,**F**,**H**,**L**).

Figure 9 shows the cyclic voltammetry (CV) responses of epoxy/graphite flake (Ep/GFs) composites with varying graphite loadings in 1 M H_2_SO_4_, including scan rate–dependent analysis (5–400 mV s^−1^) of the stabilized composites. At low GFs contents (10% and 30%), the CV curves display a quasi-rectangular shape typical of electrical double-layer capacitance (EDLC), but with low current density due to the insulating epoxy matrix and the lack of continuous conductive pathways. Composites with 50% and 70% GFs maintain the EDLC profile while showing higher current densities, attributed to the formation of a well-connected three-dimensional conductive network and sufficient porosity. This behavior is directly related to the mesoporous structure evident in SEM images, in which interconnected pores enhance electrolyte penetration and optimize the effective electrochemically active surface area.

At 80% GFs, electrochemical performance deteriorates, indicated by peak broadening, increased hysteresis, and reduced capacitive elongation, likely caused by excessive aggregation, pore blockage, and non-functional channel formation, further exacerbated by oxidation [28].

After stabilization, all samples exhibit improved rectangular shapes and reduced hysteresis, reflecting enhanced interfacial charge transport. Composites with 10–30% GFs retain near-ideal rectangular shapes even at 400 mV s^−1^, demonstrating low ohmic losses and high ionic diffusion. At low scan rates (5–20 mV s^−1^), redox peaks appear in composites with ≥30% GFs, arising from proton-coupled Faradaic reactions (C=O + H^+^ + e^−^ ⇌ C–OH), which decrease in intensity at scan rates above 100 mV s^−1^ due to diffusion limitations. This behavior is consistent with XPS analyses of similar epoxy/graphite systems, which confirmed the presence and relative proportions of redox-active functional groups [4].

Three charge storage regimes are identified: low scan rates (5–50 mV s^−1^) dominated by EDLC with full pore accessibility, intermediate rates (100–200 mV s^−1^) showing semicircular distortions from combined surface redox and ion diffusion, and high rates (400 mV s^−1^) with elliptical curves reflecting limited conductive pathways [29]. For 50–70% GFs, redox peaks persist at high scan rates, indicating continuous conductive channels, whereas at 80% GFs, severe agglomeration and pore blockage reduce electrolyte penetration, increase charge-transfer resistance, and compromise overall electrochemical performance [30].

**Figure 10:**
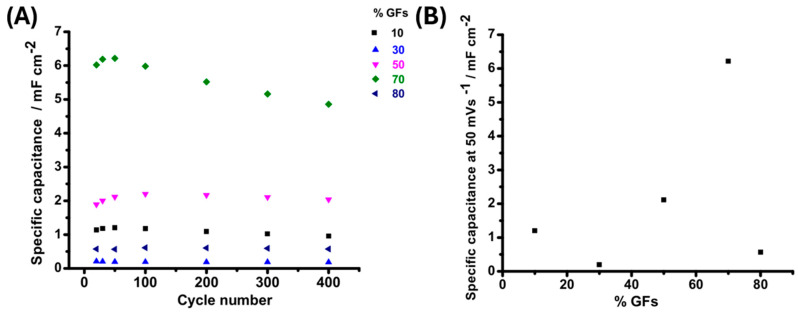
(**A**) Comparative specific capacitance of EP/GFs samples at different scan rates and (**B**) Specific capacitance at 50 mV s^−1^ as a function of GFs fraction.

Figure 10A illustrates the cycling stability of epoxy/graphite flake (Ep/GFs) composites over 400 consecutive charge–discharge cycles, highlighting the effect of filler loading on performance retention. Composites with low GF contents (10–30 wt.%) show a sharp decline in specific capacitance within the first 100 cycles, with losses up to 40%, due to insufficient formation and degradation of conductive networks at poorly integrated matrix–filler interfaces. In contrast, the 50 wt.% GFs composite demonstrates superior stability, retaining over 90% of its initial capacitance after 400 cycles. Higher loadings (70–80 wt.%) cause continuous performance degradation, with total capacitance losses of 35–50%, primarily due to particle agglomeration and progressive pore blockage that impede ion transport.

Although Raman spectroscopy was not conducted in the present study, the analysis of percolation behavior and filler dispersion is sufficiently supported by XRD, SEM, and electrochemical characterizations, which consistently demonstrate the formation of conductive networks and the strong dependence on graphite flake loading. Similar epoxy/graphite systems reported in the literature show that electrical and electrochemical trends correlate reliably with filler concentration and percolation behavior, even without Raman analysis. Refs. [3,5], supporting the structural interpretations presented here.

Likewise, although BET or methylene blue surface-area measurements were not performed, the roles of porosity, pore accessibility, and filler agglomeration were effectively evaluated through combined SEM observations and electrochemical responses. These analyses reveal clear relationships between graphite content, microstructural evolution, and ion-transport behavior, including changes in the capacitive response and hysteresis. These trends are consistent with previously reported epoxy/graphite composites, in which filler concentration has been shown to influence porosity and the effective electrochemically active surface area strongly. Consequently, the conclusions regarding pore blockage and accessibility in the present study are well supported by both experimental evidence and established literature [7].

Figure 10B shows the specific capacitance at a scan rate of 50 mV s^−1^ as a function of GFs loading. Low filler contents (10–20 wt.%) yield capacitance values below 0.6 mF cm^−2^, indicating poor electrical connectivity and insufficient percolation networks. At 30 wt.%, a moderate increase is observed, reflecting the formation of partial conductive pathways and improved access to internal surfaces. The highest performance occurs at 70 wt.%, with capacitance reaching approximately 6.5 mF cm^−2^, attributed to enhanced electrical pathways and the large surface area of the dense filler network. At 80 wt.%, specific capacitance decreases due to excessive filler loading, agglomeration, and pore collapse, which limit ion accessibility and hinder charge storage [31]. Notably, the 50 wt.% GFs composite achieves balanced performance, with a specific capacitance of ~2 mF cm^−2^, representing an optimal compromise among conductivity, porosity, and structural integrity, making it suitable for stable energy storage applications.

### 4.3. Comparison with Similar Work

Comparison of the current study with prior similar works reveals unique contributions in the field of epoxy composites reinforced with graphite flakes (EpGFs) for energy storage applications, distinguished by high filler loadings (10–80 wt.%) and comprehensive electrochemical analysis. In contrast to Darabut et al. [32], who investigated the impact of graphite types (natural, synthetic, expanded) on electrical and thermal conductivity in epoxy at low concentrations (percolation thresholds of 2.8–8.5 vol%), the present study demonstrates superiority at high loadings (50–70 wt.%) through the formation of stable three-dimensional conductive networks across 30–170 °C, achieving a specific capacitance up to 6.5 mF/cm^2^ and >90% cyclic stability, addressing limitations of low loadings in effective electrochemical accessibility.

Regarding Kumar et al. [33], who examined epoxy/graphite flake composites as thermal adhesives with XRD and FTIR, confirming structural formation and improved thermal properties at moderate loadings, the current work extends to integrated electrochemical analysis (CV, EDLC) alongside SEM/FTIR/XRD, validating quantum mechanical tunneling as the dominant charge transport mechanism and identifying an optimal balance that surpasses thermal focus to encompass electrochemical performance under harsh cyclic conditions, representing an advancement toward multifunctional energy storage applications.

In the context of Hashjin et al. [34], who developed a model for electrical conductivity in graphene-filled epoxy coatings (0.5 wt.% percolation threshold and 13-order conductivity increase), the present study stands out by confirming quantum tunneling across high loadings without costly graphene, with temperature-independent thermal stability and superior electrochemical performance (6.5 mF/cm^2^ vs. theoretical focus), offering a cost-effective and scalable alternative to graphene-enhanced conductive materials.

Finally, compared to Kim et al. [35], who achieved enhancements in thermal conductivity and fracture toughness using hydrophilic graphite flakes in epoxy/basalt fiber composites, the current study adds value by focusing on pure electrical and electrochemical properties without additional fibers, avoiding agglomeration at 50 wt.% to ensure effective electrolyte access, underscoring the importance of moderate loadings in balancing conductivity, mechanical stability, and energy efficiency for sustainable storage devices. In contrast to low-loading systems, where conductivity enhancement is mainly driven by percolation onset, this study reveals that a nuanced interplay among conductive continuity, pore accessibility, and interfacial integrity governs electrochemical performance at ultrahigh filler concentrations.

## 5. Conclusions

This study demonstrates that epoxy composites reinforced with graphite flakes (GFs) exhibit significant variations in their electrical, structural, and chemical properties with filler concentration. Composites containing approximately 50 wt.% GFs achieve an optimal balance of high electrical conductivity, mechanical stability, and sufficient ionic permeability. At this concentration, a well-connected three-dimensional conductive network forms while maintaining adequate porosity, facilitating efficient ion transport and charge mobility. Electrical conductivity continues to improve with graphite content up to 70 wt.%, where quantum-mechanical tunneling is identified as the dominant charge-transport mechanism, ensuring stable, temperature-independent performance across a broad range. This highlights the robustness and reliability of these composites for energy-storage applications.

However, filler loadings above 70 wt.% lead to excessive agglomeration and partial pore blockage, increasing transport resistance and reducing electrochemical efficiency. These structural changes induce microstructural defects and localized disruptions in the polymer matrix, as confirmed by SEM and FTIR analyses. Morphological and spectroscopic observations indicate a clear transition from relatively homogeneous filler dispersion at low concentrations to significant structural degradation at high loadings. Overall, a graphite flake content of around 50 wt.% provides the most favorable composite architecture, effectively balancing continuous conductive pathways with adequate electrolyte accessibility. In contrast, excessive filler incorporation compromises overall performance due to interfacial limitations and microstructural deterioration.

Future work will include BET surface-area and Raman spectroscopy analyses to quantify porosity further and the structural ordering of sp^2^ domains, providing more detailed insights into the microstructural and electrochemical behavior of these composites.

## Data Availability

The original contributions presented in this study are included in the article. Further inquiries can be directed to the corresponding author.
